# Toxigenic Species *Aspergillus parasiticus* Originating from Maize Kernels Grown in Serbia

**DOI:** 10.3390/toxins13120847

**Published:** 2021-11-26

**Authors:** Milica Nikolić, Iva Savić, Ana Nikolić, Marko Jauković, Vesna Kandić, Milan Stevanović, Slavica Stanković

**Affiliations:** 1 Maize Research Institute Zemun Polje, 11185 Belgrade, Serbia; isavic@mrizp.rs (I.S.); anikolic@mrizp.rs (A.N.); vkandic@mrizp.rs (V.K.); mstevanovic@mrizp.rs (M.S.); sstojkov@mrizp.rs (S.S.); 2 Jugoinspekt Beograd, 11000 Belgrade, Serbia; jaukovicmarko@gmail.com

**Keywords:** *Aspergillus parasiticus*, aflatoxins, maize

## Abstract

In Serbia, aspergillus ear rot caused by the disease pathogen *Aspergillus parasiticus* (*A. parasiticus*) was first detected in 2012 under both field and storage conditions. Global climate shifts, primarily warming, favour the contamination of maize with aflatoxins in temperate climates, including Serbia. A five-year study (2012–2016) comprising of 46 *A. parasiticus* strains isolated from maize kernels was performed to observe the morphological, molecular, pathogenic, and toxigenic traits of this pathogen. The HPLC method was applied to evaluate mycotoxin concentrations in this causal agent. The *A. parasiticus* isolates synthesised mainly aflatoxin AFB1 (84.78%). The percentage of isolates synthesising aflatoxin AFG1 (15.22%) was considerably lower. Furthermore, the concentration of AFG1 was higher than that of AFB1 in eight isolates. The polyphase approach, used to characterise isolates, showed that they were *A. parasiticus* species. This identification was verified by the multiplex RLFP-PCR detection method with the use of restriction enzymes. These results form an excellent baseline for further studies with the aim of application in the production, processing, and storage of cereal grains and seeds, and in technological processes to ensure the safe production of food and feed.

## 1. Introduction

Maize is one of the most important cultivated cereals in the world, but by its production, it ranks first in Serbia. On average, it is annually cultivated on the area of approximately 1,000,000 hectares (source: Statistical Office of the Republic of Serbia). This field crop meets domestic needs but is also a strategic product intended for export. Therefore, any problem related to this food and feed crop poses a great economic and health concern. Maize ear rot is caused by many pathogenic and toxigenic fungal species in Serbia. *Aspergillus flavus* Link and *Aspergillus parasiticus* Speare are the most important species causing aspergillosis kernel and ear rot in the world [1].

The *A. parasiticus* species was first identified on sugarcane in Hawaii in 1912, from where it spread over wide areas in the continental USA, but not as widely as *A. flavus* [2]. The species has also been isolated from soils and fodder in the USA, South America, southern Africa, India, and Australia; it is almost completely unknown in southeast Asia [3,4]. In soils, the populations of this species are mainly in peanuts fields in the USA, Australia [5], Uganda, [6], Argentina [7], and Botswana [8]. *A. parasiticus* has been detected to a lesser extent in cereal fields [5,9,10,11]. There appears to be a limited amount of data regarding the presence of *A. parasiticus* in field maize in the USA. In Europe, the occurrence of *A. parasiticus* in maize seems to be very rare. For example, maize research in Italy found a low incidence of *A. parasiticus* even in the temperate climate region during the growing season [12].

This may be explained by the fact that although species of the genus *Aspergillus* can be detected in all climate zones, their occurrence is more common in subtropical regions (latitude 26–35° north and south of the Equator) than in tropical and cooler temperature regions. These species are rarely present at latitudes over 45° [13]. High temperatures and relative humidity and especially high nocturnal temperatures are necessary for the appearance and intensive development of ear aspergillosis under field conditions.

In recent years, changes in climate have favoured the unusually high incidence of the pathogenic and toxigenic *Aspergillus* spp. during the maize growing season under agroecological conditions in Serbia. *A. parasiticus* was initially isolated from maize kernels under field conditions in the 2012 growing season in Serbia [10]. Very high temperatures and extreme prolonged drought conditions recorded during the vegetation period in 2012 resulted in a very low precipitation factor (0.27 to 1.45) or arid conditions. Subsequently, the mean annual temperature exceeded 12 °C, which is why the *Aspergillus* species appeared in the fields throughout Serbia in contrast to earlier years when those species were identified only in storage. This was the first incidence of *A. parasiticus* isolates obtained from maize [14], wheat [10], and barley kernels [11] cultivated under Serbian climate conditions.

In addition to ear rot, the deleterious effects of *A. parasiticus* are also reflected in its ability to synthesise secondary metabolites, mycotoxins, which negatively affect human and animal health. Aflatoxins are the most toxic and carcinogenic naturally produced toxins [1,15]. They pose a health threat to humans due to high concentrations in contaminated maize, cotton, soybeans, peanuts, and nutlets prior to harvest [16]. In addition, soon after the ingestion of aflatoxin B1 through food, it is transformed into aflatoxin M1 and excreted into milk in mammals. Aflatoxins cause carcinogenic, mutagenic, teratogenic, and immunomodulatory changes in humans and animals [17]. Until rather recently, aflatoxins have not been a matter for concern in primary production in Europe [18]; however, 2012 marked a significant year for Serbia in terms of extreme levels of mycotoxin contamination found in their maize [19].

The current study offers the first comprehensive report of the presence of *A. parasiticus* in maize kernels under field conditions in Serbia and other countries of the Western Balkans. The objective of this study was to examine this toxigenic pathogen through detailed identification and characterisation in order to determine the prevalence and toxicity of this pathogen as well as its threat potential to food and feed safety in Serbia and surrounding countries in Europe.

## 2. Results

The collected maize ears exhibited dark green rot lesions, which are a typical ear rot symptom. The size of the observed lesions ranged from small, barely noticeable lesions to almost completely decayed ears covered with green spores. Dark green spores developed on the lesion surface, starting from the infection site.

The pathogenicity of the isolates was confirmed by the artificial inoculation of maize ears. Disease symptoms caused by fungi appeared in all of the inoculated ears, and these symptoms were the same as those observed on the ears collected from different locations. The incidence of the disease caused by fungal pathogen in inoculated maize ears was 100% in each of the four replicates. No symptoms were observed on the negative control ears. The morphological characteristics of the isolates from the inoculated ears were identical with the original isolates, which confirmed the pathogenicity of the isolates and fulfilled Koch’s postulates, which determine a causal relationship between an organism and a disease.

**Figure 1:**
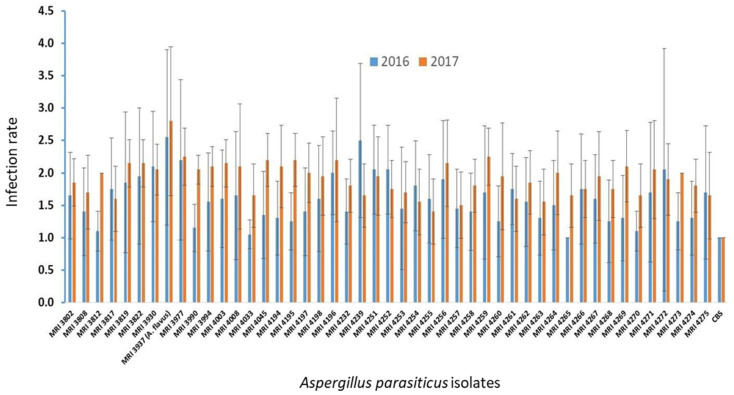
Assessment of pathogenicity of *A. parasiticus* isolates on maize hybrids. Legend: the vertical bar indicates the standard error of the mean.

Under conditions of artificial inoculation, during the first trial year (2016), the tested isolates exhibited an average degree of the disease intensity in the range of 1–2.5. The lowest, i.e., highest pathogenicity was observed in the isolates MRI 4265 (average degree: 1.2), i.e., MRI 4239 (average degree: 2.5), respectively. IsolateMRI 3937 (*A. flavus*) was used as a positive control in the test (Figure 1).

The average assessment ratings of the infection intensity in the second year of the study, 2017, ranged from 1.4 to 2.25. The lowest and highest pathogenicity was observed respectively in the following isolates: MRI 4255, and MRI 3937 (*A. flavus*), MRI 3977 and MRI 4259, (Figure 1).

Comparison of the average degrees of infection between the two years (average degree for 2016: 1.59; average degree for 2017: 1.89) did not exhibit a consistent pathogenicity.

### 2.1. Macroscopic and Microscopic Morphological Characteristics of Identified Isolates

Twenty-four hours after culturing, all *A. parasiticus* isolates on PDA formed a rudiment of a white, aerial mycelium about 3–4 mm in diameter. After 7-day growth on PDA at 25 °C, the *A. parasiticus* isolates exhibited relatively uniform macroscopic traits (appearance, colour, and the growth of the colony). The isolates formed an abundant, dark green aerial, dense mycelium of cottony appearance. The *A. parasiticus* colonies that formed on the PDA were flat and velvety, ivy green with wooly centres. The central zone of the colonies was olive, greenish grey, and the borders were yellowish. In some isolates, the colony pigmentation was reversed. A spore mass was formed on the surface of each colony. The reference isolate CBS100926, originating from the Netherlands, formed a colony similar in appearance to the isolates originating from Serbia.

On the CYA medium, the mycelium was similar but more dense, and on the MEA medium, the mycelium had concentric rings of dark green spores, which was not observed on the other two media.

The average growth after the 7-day incubation on PDA was in the 41.25–73.50 mm range; on the CYA medium, it ranged between 36.5 and 69.25 mm, and on the MEA medium, it ranged between 40.63 and 62.25 mm.

**Figure 2:**
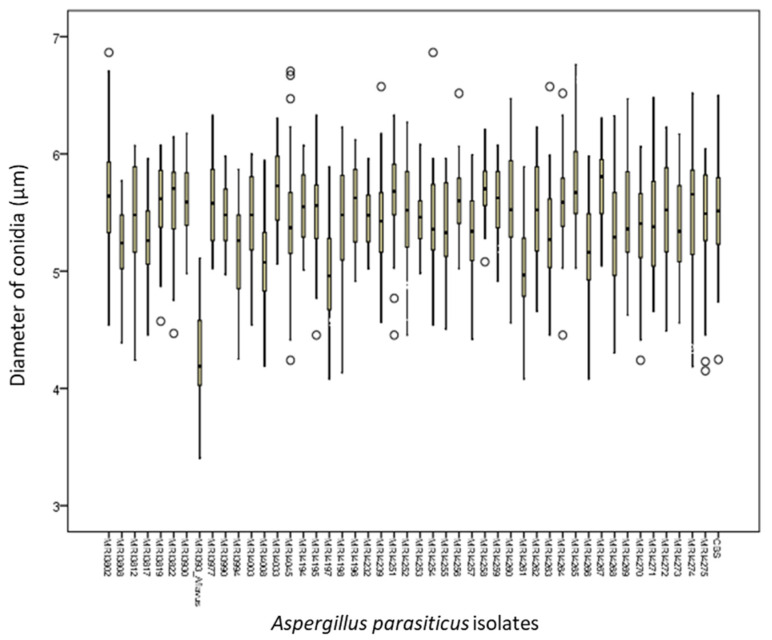
Diameter of conidia (µm) of all isolates *A. parasiticus* originating from MEA growth media. Legend: the vertical bar indicates the standard error of the mean.

The size of spherical, denticulate, thick-walled conidia of all investigated isolates ranged from 3.41 to 6.86 µm. Figure 2 is a comparative presentation of the average diameters of conidia of *A. parasiticus* observed under the microscope. *A. parasiticus* isolates varied in the diameters of conidia. The MRI 3937 (*A. flavus*) isolate, used as a positive control, formed conidia with the smallest diameter (4.27 µm). Conidia with the smallest average diameter (4.95 mm) were formed by the MRI 4197 isolate, while conidia with the largest average diameter (5.79 µm) were formed by the MRI 4265 isolate. The reference isolate CBS100926 formed conidia with the average diameter of 5.49 µm (Figure 2).

The observed isolates differed in terms of the formation of metulae, while all isolates formed phialides and conidia. Globular vesicles had a diameter of 14–25 µm.

### 2.2. Morphology of Sclerotia

Fourteen days after culturing on the CZ substrate, sclerotia, black, globular masses, composed of densely interwoven hyphae, were observed in mycelia of certain isolates.

Based on the average size, the largest sclerotia (1168.6 µm) was formed by the MRI 3990 isolate, while the smallest (517.9 µm) was formed by the MRI 3937 (*A. flavus*) isolate, which was used as a positive control. The smallest sclerotia were formed by the MRI 4198 (549.3 µm) isolate. The diameter of sclerotia varied from 395.2 µm (MRI 4194) to 1550.6 µm (MRI 3990). The reference isolate (CBS100926) did not form sclerotia.

**Figure 3:**
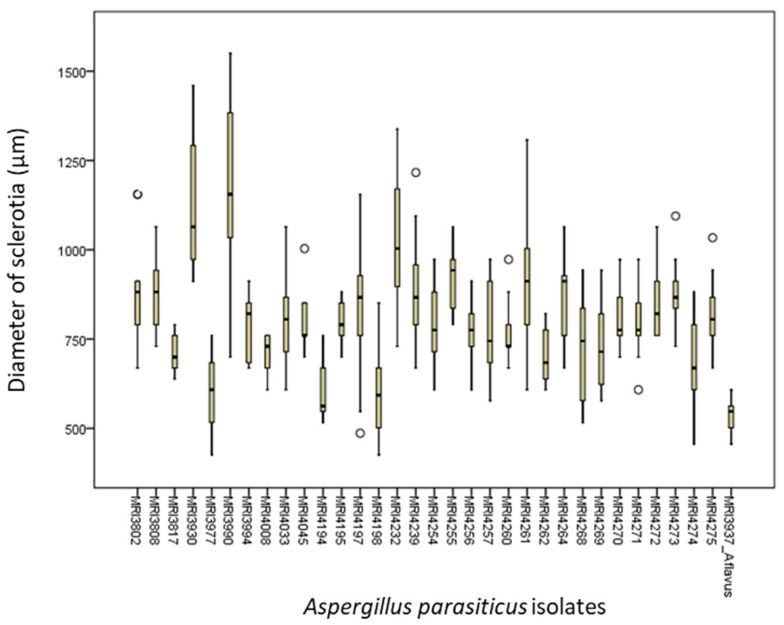
The diameter of sclerotia of *A. parasiticus* isolates on the CZ substrate. Legend: vertical bars present a standard error of mean.

After 14-day incubation during the monitoring of the appearance and growth of the colonies on the CZ substrate at 30 °C, scelorotia were observed in a great number (73%) of isolates, with the remaining isolates not forming any sclerotia (Figure 3). Only the MRI 4033 isolate formed sclerotia after five days of incubation.

### 2.3. Molecular Identification of Aspergillus parasiticus Isolates

#### 2.3.1. RFLP Analysis of ITS rDNA Region

A total of 46 *A. parasiticus* isolates and the reference isolate CBS100926 were used for the RFLP-PCR analysis. The *A. flavus* isolate, MRI 3937, served as a positive control.

As a result of the polymerase chain reaction, expected nucleic acid fragments of size of 674 bp were amplified by using IGS-F/IGS-R primer, according to the protocol described previously [20]. The 674 bp fragments, which are specific to *A. flavus* and *A. parasiticus*, were amplified in 48 samples.

PCR products obtained by the multiplication of the primer pair IGS-F/IGS-R that amplifies the intron gene region for glutamine synthetase (GS) were treated with the *Bgl*II restriction enzyme. The RFLP analysis of the products in the agarose gel yielded restriction profiles that clearly showed differences among analysed samples.

The virtual RFLP analysis of ITS sequences of the rDNK region obtained with the *Bgl*II restriction enzyme reveal two restriction sites for this enzyme in *A. flavus* isolates originating from Serbia, while sequences of *A. parasiticus* isolates had one restriction site.

After digestion with the *Bgl*II enzyme, the expected fragment sizes in *A. flavus* were 102 bp, 210 bp, and 362 bp, and in *A. parasiticus*, they were 311 bp and 363 bp. The RFLP analysis with the *Bgl*II enzyme of the IGS-F/IGS-R PCR products of selected *A. parasiticus* and *A. flavus* isolates confirmed the results of the virtual RFLP analysis.

After digestion with the *Bgl*II restriction enzyme, all tested *A. parasiticus* isolates had mutually identical electrophoretic profiles, which were different from the *A. flavus* isolate. Amplified 363 bp and 311 bp fragments were detected in all samples identified as *A. parasiticus*, including the reference *A. parasiticus* isolate (CBS100926), while the of sizes 102 bp, 210 bp, and 362 bp fragments were amplified in the *A. flavus* isolate. No amplification occurred in the negative control. The multiplex PCR detection confirmed the identity of all isolates previously characterised on the basis of the morphological, ecological, breeding, and toxigenic traits, as the *A. parasiticus* species.

#### 2.3.2. Toxicological Profile of *Aspergillus parasiticus* Isolates

The variability of aflatoxins B1, B2, G1, and G2 across all isolates was determined by quantitative and qualitative analysis of the production potential results by high-performance liquid chromatography with fluorescence detection (HPLC-FLD).

The average synthesis of aflatoxin B1 was 4385.63 µg/kg by which the isolates of this group were classified into strong producers of AFB1. The average synthesis of aflatoxin B2 was 559.47 µg/kg, with the corresponding values for aflatoxin G1 and G2 averaging 3880.12 µg/kg, respectively. The average synthesis of aflatoxin G2 amounted to 168.65 µg/kg.

The highest and lowest concentrations of AFB1 were detected in the MRI 4194 (7361.03 µg/kg) and MRI 3808 (14.24 µg/kg) isolates, respectively. The highest concentrations of AFG1 were determined in MRI 4198 (7421.58 µg/kg), MRI 4195 (7191.62 µg/kg), and MRI 4197 (7122.59 µg/kg). On the other hand, the lowest concentrations of this mycotoxin were detected in the MRI 3808 (3.27 µg/kg) isolate (Table 1).

The synthesis of AFG2 did not occur in MRI 4194, MRI 4252, and MRI 4265, while MRI 3808 and MRI 4275 were not able to synthesise AFB2 and AFG2 (Table 1).

Nevertheless, it was established that eight isolates synthesised AFG1 in higher concentrations than AFB1.

A great percentage (50%) of analysed isolates did not synthesise aflatoxins B1, B2, G1, and G2, or they synthesised them in very low concentrations.

Based on the obtained results, a great diversity in the production of all individual aflatoxins was observed.

The results obtained by the HPLC method indicated a statistically highly significant correlation between the production potentials of AFB1 and AFG1 in *A. parasiticus* isolates (r = 0.82 **) as well as between the production potentials of AFB2 and AFG2 (r = 0.63 **).

## 3. Discussion

Our research showed an average growth of *A. parasiticus* isolates after 7 days of incubation at 25 °C on PDA medium to be in the range 41.25–73.50 mm range, on the CYA medium, the growth range was 36.5–69.25 mm, and on the MEA medium, it was 40.63–62.25 mm. The colonies were a dark green colour with yellow edges and displayed copious spore mass. Similar observations were made by others [21,22] regarding the colour of *A. parasiticus* colonies and robust growth on various media, with diameters reaching 70 mm. Some authors [23,24] recorded good growth of *A. parasiticus* colonies at temperatures reaching up to 42 °C, indicating the broad temperature range in which this fungus can thrive.

Pitt and Hocking described microscopic morphological traits of *Aspergillus* species in great detail [25]. They noted that conidiophores bore subsurface or surface hyphae. The length of stipes ranged from 250 to 500 µm. Their walls were colourless or pale brown and smooth. The diameter of spherical vesicles varied from 20 to 35 µm. They were fertile over ¾ of the surface and mostly bore only phialides. In some isolates, up to 20% of heads also bore metulae. The length of phialides ranged from 7 to 11 µm. Conidia were spherical, approximately 4.0–6.0 µm in diameter with distinctly roughened walls. The authors concluded that the appearance and shape of stipes, vesicles, and phialides, the presence or absence of metulae, and the size and the shape of spores are less reliable indicators in the identification of *Aspergillus* species. However, conidium wall ornamentation is a reliable criterion for distinguishing morphologically similar species. *A. parasiticus* have spherical conidia with relatively heavy spiny or bristly thick walls. Vesicle diameters of *A. parasiticus* rarely exceed 30 µm, and metulae are not common.

The present study included microscopic morphological traits of isolates from the 7-day cultures grown on the MEA medium. By making nutritive preparations and observing them under a binocular and a light microscope, special attention was given to the determination of spherical, dented conidia, detected in all tested isolates. In agreement with the literature, the diameter of vesicles did not exceed 30 µm in any of the isolates. Interestingly, some (15%) *A. parasiticus* isolates originating from Serbia produced metulae and phialides, which is also in accordance with the values described in the literature for *A. parasiticus* [26]. Baquião and others [27] recorded serrated, spherical conidia with thick walls and the presence of vesicles with phialides but also with phialides and metulae. However, minimum and maximum values of measured diameters deviated, in some cases, from extreme values of conidium diameters (3.41–6.86 µm). The average conidium diameter of *A. parasiticus* isolates originating from Serbia ranged from 4.95 to 5.79 μm on the MEA medium (aver 5.43 μm), which is in agreement with existing literature for this species. These dimensions of conidia are similar to the average conidium dimensions (4.4 μm) of *A. parasiticus* isolates reported earlier [10]. According to studies of *A. parasiticus* isolated from wheat carried out by Dovičičova and her colleagues [23], stipes were dented, vesicles were spherical and fertile in the upper half or alongside the entire surface, mainly with phialides only (9.5 µm) and serrated conidia (4.9–5.2 µm). Nyongesa and others [28] reported the presence of metulae and phialides, vesicle diameters of 24–30 µm, and conidium diameters of 4–5.8 µm in *A. parasiticus* isolates from maize samples in Kenya.

Pitt and Hocking [25] reported that sclerotia were occasionally produced in *A. parasiticus* and that they were white at first and later became black; that they were spherical and 400–800 µm in diameter. The majority of sclerotia produced exceeded maximum and minimum values (395.2–1550.6 µm). Based on the average diameter, the greatest and smallest sclerotia were produced by the MRI 3990 isolate (1168.6 µm) and the MRI 4198 isolate (549.3 µm), respectively. It was observed that many of the isolates (73%) produced sclerotia. In Italy, studies [12,29] reported results that were similar to our present results where up to 80% of *A. parasiticus* isolates produced sclerotia on the CZ medium at 30 °C. In Portugal, [21] established that 52.6% of tested *A. parasiticus* isolates had produced sclerotia. Okoth and others [30] observed the production of sclerotia in *Aspergillus* species originating from the Nandi county with a diameter range of 935–1590 µm but also unusually small sclerotia (240–280 µm) from the Makueni county in Kenya. Frisvad and colleagues [22] determined the production of large sclerotia in the majority of *A. flavus* isolates tested on the CYA medium at both 25 °C and 37 °C. Similar results were gained in the present study, except for the fact that sclerotia were produced at 30 °C, and the most of our isolates were *A. parasiticus.*

The PCR-RFLP is reliable method for the rapid and accurate identification of *A. parasiticus* isolates. Khoury and others [20] applied the PCR-RFLP method for the molecular separation of isolates from a pure culture and proved the existence of two species, *A. flavus* and *A. parasiticus*. Ahmad and colleagues [31], using this method, detected and differentiated *A. flavus* and *A. parasiticus* isolates originating from peanuts. Nikolić and others [10] worked on a polyphasic approach to the identification of isolates originating from wheat kernels and established the presence of *A. parasiticus* by the application of the PCR-RFLP method.

This study also encompassed the determination of the synthesising ability (qualitative and quantitative analysis) for certain aflatoxins (B1, B2, G1, and G2) in *A. parasiticus* isolates using the HPLC-FLD method. The gained results indicate that there were significant differences in the average production of AFB1 and AFG1 compared to AFB2 and AFG2. The average concentration of aflatoxin B1 amounted to 4385.63 µg/kg, which classifies the isolates of this species into strong AFB1 producers. The average synthesis of aflatoxin B2 was 559.47 µg/kg. The average synthesis of aflatoxin G1 was 3880.12 µg/kg, which classifies them into strong AFG1 producers, while the average synthesis of aflatoxin G2 was 168.65 µg/kg. It was observed that aflatoxin B1 was a dominant aflatoxin of *A. parasiticus* isolates in Serbia, while aflatoxin G1 was recorded in a significantly lower percentage of isolates (84.78% vs. 15.22%). The AFG2 synthesis did not occur in MRI 4194, MRI 4252, and MRI 4265 isolates, whereas MRI 3808 and MRI 4275 isolates were not capable of synthesising AFB2 and AFG2.

Donner and colleagues [32] have also observed the ability of *A. parasiticus* isolates to synthesise aflatoxins and reported the average concentrations of AFB1 and AFG1 to be 90–2092 µg/kg and 99–450 µg/kg, respectively. The range of concentrations of synthesised AFB1 and AFG1 in these studies was 90–4957 µg/kg and 0–6131 µg/kg, respectively. In Portugal [33], studies using contaminated almond and chestnut samples established that the majority of observed isolates (86%) showed a typical toxicological profile of *A. parasiticus*. Thus, they are strong producers of aflatoxins B1 and G1, while there was no CPA production. However, Rodrigues [33] also showed that eight isolates (4%) did not synthesise aflatoxins of the G group, as well as that 7% isolates did not synthesise aflatoxin G1 at concentrations higher than aflatoxin B1. During the investigation of the production potential of total aflatoxins in *A. parasiticus* isolates originating from Nandi county in Kenya, Okoth and associates [30] detected significantly higher ranges of concentration variation, from 9982 to 13,662 µg/kg. In the same research, the production potential of individual aflatoxins ranged as follows: 603–11077 µg/kg (AFB1), 208–2876 µg/kg (AFB2), 24–5982 µg/kg (AFG1), and 0–7798 µg/kg (AFG2). According to the production potential of the isolates originating from the Makueni county, the values of the synthesised total (22–55,419 µg/kg) and individual aflatoxins AFB1 (22–15,139 µg/kg), AFB2 (0–3141 µg/kg), AFG1 (0–28,728 µg/kg), and AFG2 (0–8411 µg/kg) were much higher. The same authors [30] have not determined differences in the increased synthesis between AFG1 and AFB1.

Baquião and others [27] have established the average concentrations of synthesised individual aflatoxins in nutlets in Brazil: AFB1 6172 µg/kg, AFB2 184 µg/kg, AFG1 16.776 µg/kg, and AFG2 313 µg/kg. All except two isolates have synthesised aflatoxins of B and G groups, while the stated two have synthesised only aflatoxins of the B group.

Nikolić and colleagues [34] compared the toxigenic potential of *A. flavus* and *A. parasiticus* from maize and have established by the HPLC-FLD method that the concentrations of individual aflatoxins of the group G ranged from 8.03 to 7421.58 µg/kg for AFG1 and from 0 to 395.18 µg/kg for AFG2. In the later study, the same authors [10] examined the production potential of *A. parasiticus* isolates originating from wheat kernels harvested under different agroclimatic conditions in Serbia and determined that all the isolates were capable of synthesising aflatoxins in the following concentration ranges: 4859.15–7361.03 µg/kg (AFB1), 142.81–1543.8 µg/kg (AFB2), and 3085.11–7191.62 µg/kg (AFG1). One isolate did not synthesise AFG2, while all the remaining ones did, in the range of 86.92–181.76 µg/kg. However, the authors also determined that two isolates synthesised AFG1 in concentrations higher than AFB1.

Baranyi and others [35] reported that all *A. parasiticus* isolates produced higher amounts of aflatoxin G1 than aflatoxin B1. Similar results were reported in previous studies [36,37,38]. The results gained from these studies are different from our results where only eight isolates synthesised AFG1 in higher concentrations than AFB1.

Rodrigues [33] reported the existence of isolates that did not synthesise aflatoxins. The author identified four different chemotypes in *A. parasiticus* isolates. The majority of tested isolates (81%) showed a typical profile of *A. parasiticus*, that is, a high synthesis of aflatoxins B and G. Moreover, only 4% of isolates did not produce aflatoxins G, and their chromatogram showed peaks similar to those of *A. flavus*. Only 7% of isolates showed atypical results, such as a higher synthesis of aflatoxin G than aflatoxin B. While interesting, the *A. parasiticus* in her study was extracted from almonds and chestnuts in Portugal, which may explain why the results significantly differ from results obtained in our study based on maize samples. Others [39] also noted that 3–6% of naturally occurring *A. parasiticus* isolates from peanuts had no ability to synthesise aflatoxins. The investigations carried out within the present study indicate that there is a high percentage (50%) of tested isolates, which did not synthesise aflatoxins B1, B2, G1, and G2 or did in very low concentrations below the detection threshold. The existence of atoxigenic isolates of this species may represent a significant step forward for Serbia, as native atoxigenic strains have been increasingly used in the biological control of virulent fungal strains around the world.

The study revealed for the first time, the existence of *A. parasiticus* on maize kernels in Serbia. Presently, there is very little data on the distribution and diversity of the species. Considering the effects of ongoing climate changes, a more intensive presence of the mentioned species was to be expected on maize kernels in Serbia and generally, in Europe. This was confirmed by the obtained results. The revelation of the high incidence of atoxigenic *A. parasiticus* isolates may offer a potential mitigation solution.

## 4. Conclusions

*A. parasiticus* was studied over a five-year period (2012–2016), for the first time in Serbian maize, as a cause of ear aspergillosis rot in fields and warehouses. Morphological, pathogenic, ecological, breeding, toxigenic, and molecular characterization of *A. parasiticus* isolates originating from maize grains collected in different parts of the country provided insight into the presence, distribution, and biodiversity of this species in Serbia. Since *A. parasiticus* isolates that do not synthesize aflatoxins have also been identified by the results in this study, the possibility of studying atoxigenic isolates and their use as biological agents in plant protection should be considered.

## 5. Materials and Methods

### 5.1. Sample Collection and Fungal Isolation

During the 2012–2016 period, maize ear samples were collected from approximately 70 locations from different districts in the territory of the Republic of Serbia. Plants with dark green powder coat on the ears and maize kernels were used as the source material for pathogen isolation. Isolation of the fungi was performed by placing decayed kernels on potato dextrose agar (PDA) in Petri plates. Following incubation at 25 °C for 5 days, plates were examined. Fragments of fungal colonies were transferred to sterile PDA and were incubated for 7 days. Monospore cultures were obtained and preserved on PDA, malt extract agar (MEA), and Czapek yeast agar (CYA) slants in the refrigerator at 4 °C. A total of 46 isolates from different geographical regions were chosen for further analyses. The MRI 3937 isolate (*A. flavus*) from Maize Research Institute collection and a reference *A. parasiticus* (CBS 100926) isolate from the collection CBS Culture Collection of Fungi were used as positive control.

### 5.2. Pathogenicity Test

A method developed by Reid´s group [40] was used for artificial inoculations. Healthy maize hybrids were used to assess pathogenicity in field trials during two growing seasons (2016–2017) and to fulfil Koch’s postulates, which verifies the cause and effect between an organism and a disease. A selected group of 20 hybrids was inoculated by the injecting fungal spore suspension into the silk channel. PDA (Biolife, Milan, Italy) was used to culture *A. parasiticus* isolates at 25 °C in the dark. The spore suspension was prepared by flooding the 7-day-old mycelia with 10 mL of sterile distilled water and 0.01% Tween 20. The haemocytometer was used to adjust the suspension concentration to approximately 3 × 10^7^ spores mL^−1^. Inoculation was carried out three days after 50% of plants reached the silking stage. Two mL of inoculum was injected through the silk channel on the upper ear. Positive control ears were inoculated by applying the equal amount of *A. flavus* inoculum and negative control ears by applying the equal amount of sterile distilled water. Five ears in four replicates were inoculated with the conidial suspension (a total of 20 ears per isolate). Visual rating was performed on the 1–7 scale (1—complete absence of symptoms; 7—76–100% infected kernels), as described previously [40]. The pathogen was reisolated anew from the wounds that developed on inoculated ears.

### 5.3. Morphological Characterization

Isolates were three-point inoculated on PDA, MEA, and CYA in Petri plates and incubated in the dark at 25 °C. Macroscopic features were observed on PDA and CYA medium after seven days of incubation. Colony growth rates and appearance, reverse colours of mycelia, and production of sclerotia were recorded after 7 days. Four plates per medium were incubated for each isolate. During the incubation period, colony growth was measured daily and presented as average growth (mm/day). Microscopic slides were prepared from 7-day-old cultures grown on MEA. The morphology of conidia, vesicles, and phialides, the presence or absence of metulae, and the dimension, shape, and texture of conidia were assessed after 7 days. Conidial measurement was performed by observing 30 randomly selected conidia from cultures of the seven-day-old isolates using a Carl Zeiss Axiolab A1 light microscope. The results obtained were statistically processed by one-way analysis of variance (ANOVA) according to a completely randomized plan with 30 conidia per isolate. Using Duncan’s post hoc test, the significance of the difference between the means was determined at 0.05 of α.

The occurrence of sclerotia was monitored on a CZ substrate in 14-day-old cultures maintained in the dark at 25 °C. The sclerotia were washed in 96% alcohol and visualized under a microscope. The diameter of the sclerotia was determined by averaging 30 repeated measurements.

### 5.4. Molecular Analysis

#### 5.4.1. DNA Extraction

A fungal mycelium was obtained from isolates grown on Czapek yeast extract agar (CYA) for 2 days at 25 °C. DNA extraction from fungal strains was performed according to an established protocol [41]. Total DNA was resuspended in 30 µL of deionised H_2_O and stored in the freezer at −20 °C until use.

#### 5.4.2. PCR Amplification

The intergenic spacer (IGS) for the AF biosynthesis genes *aflJ* and aflR was used as a target in order to distinguish between *A. flavus* and *A. parasiticus*. The available published regions from the isolates were amplified by a designed primer pair IGS-F/IGS-R [24,42] that corresponded to a PCR product of 674 bp [20]. The sequences of the primers used were as follows: IGS-F, 5-AAGGAATTCAGGAATTCTCAATTG-3; IGS-R, 5-GTCCACCGGCAAATCGCCGTGCG-3.

The amplification of fungal DNA was performed in a total reaction volume of 50 µL, containing reaction buffer, 1.5 mM MgCl_2_, 0.8 mM each dNTP (dATP, dCTP, dGTP, and dTTP), 1 unit of *Taq* DNA polymerase, 100 ng template DNA, and 0.5 µM of each primer. Amplifications were done with the following cycling parameters: initial denaturation at 94 °C for 4 min; 35 cycles of denaturation at 94 °C for 40 s, annealing at 58 °C for 40 s and extension at 72 °C for 1 min; and a final extension at 72 °C for 10 min. The PCR products were separated by agarose gel electrophoresis and stained with ethidium bromide. PCR amplification of the IGS region yielded a 674 bp band.

#### 5.4.3. Restriction Site Analysis of PCR Products

The analysis of the IGS sequence indicated that the restriction enzyme *Bgl*II cleaved the PCR products into fragments that are useful tools for the detection of *A.* *flavus* and *A. parasiticus* [19].

Following amplification, the PCR products were digested with the restriction enzyme *BglII* (Roche, Germany). The reactions were performed in a total volume of 40 µL containing 15 units of enzyme, 4 µL of buffer, 15 µL of the PCR product, and ultrapure water up to 40 µL. The reaction mixture was incubated at 37 °C for 1 h. Then, digested fragments were separated by electrophoresis on a 2% *w*/*v* agarose gel at 80V for 2.5 h and visualized under UV transillumination. The sizes of the resulting DNA fragments were estimated in comparison with a commercial 100 bp DNA ladder.

### 5.5. Aflatoxins Production

#### 5.5.1. Sample Preparation for the Mycotoxin Assessment

Forty-six *A. parasiticus* isolates were evaluated for levels of aflatoxin contamination. The aflatoxin production was determined in isolates inoculated in the centre of the Petri plate with a dense conidial suspension and grown on PDA as single colonies. The incubation of cultures was done at 28 ± 1 °C in the dark for 5 days [43].

#### 5.5.2. HPLC, Extraction, and Quantitation of Aflatoxins

According to a previously reported procedure [44], the contents of each PDA plate were collected into a tube as fungal biomass, which was placed in glass vials of a known weight and reweighed. The fungal biomass was extracted with an acetonitrile–water (90:10, *v*/*v*) solvent mixture in the ratio of 100:1, *v*/*m*. A reciprocal shaker was used to shake vials for 30 min at high speed. One mL aliquot of each extracted isolate was centrifuged at 12,000× *g* for 10 min. The HPLC–fluorescence detection method was used to confirm the presence of aflatoxins in the supernatant [44]. Data are reported as the mean value of three independent injections.

## Figures and Tables

**Table 1:** Toxicological profile of *Aspergillus parasiticus* isolates originating from maize.

Isolate	AFB1 µg/kg	AFB2 µg/kg	AFG1 µg/kg	AFG2 µg/kg
3802	3429.82	544.25	3461.35	260.47
3808	14.24	×	3.27	×
3812	×	×	×	×
3817	×	×	×	×
3819	×	×	×	×
3822	×	×	×	×
3930	3438.01	331.92	3437.81	306.65
3937 *(A. flavus)*	646.05	0.27	12.04	×
3977	911.03	561.35	554.22	47.69
3990	5698.47	303.94	6518.88	90.52
3994	×	×	×	×
4003	×	×	×	×
4008	×	×	×	×
4033	×	×	×	×
4045	6716.42	1543.80	5189.43	181.76
4194	7361.03	995.41	3085.11	×
4195	6431.77	287.25	7191.62	110.11
4197	5850.87	527.31	7122.59	395.18
4198	6741.07	652.58	7421.58	374.93
4196	4409.10	143.97	5357.14	68.92
4232	5708.12	142.81	6495.07	86.92
4239	×	×	×	×
4251	5918.46	254.03	6606.65	98.25
4252	6816.08	721.28	2166.36	×
4253	15.46	×	×	×
4254	×	×	×	×
4255	89.67	×	×	×
4256	×	×	×	×
4257	×	×	×	×
4258	6884.01	1.590,6	2946.52	10.43
4259	×	×	×	×
4260	×	×	×	×
4261	5198.68	403.79	5.13	128.02
4262	5776.73	629.68	5746.97	217.83
4263	×	×	×	×
4264	×	×	×	×
4265	5968.77	1733.52	634.79	×
4266	5168.79	351.97	5157.56	119.18
4267	×	×	×	×
4268	×	×	×	×
4269	×	×	×	×
4270	×	×	×	×
4271	×	×	×	×
4272	×	×	×	×
4273	×	×	×	×
4274	6029.33	500.80	6240.54	201.47
4275	33.05	×	8.03	×

×—samples containing very low concentrations that were outside the range of used Mycoseb columns.

## Data Availability

Data is contained within the article.
